# Celebrating the life of Professor Felix Tietche

**DOI:** 10.11604/pamj.2016.24.65.9062

**Published:** 2016-05-16

**Authors:** Francois-Xavier Mbopi-Keou

**Affiliations:** 1 Member of the UNAIDS Strategic & Technical Advisory Committee and University of Yaounde I, Cameroon

**Keywords:** Professor Felix Tietche, Celebrating the life

## Obituary

Exactly 8 years ago, on 16 May 2009, died one of the finest scientists in the field of pediatric in Cameroon.

Professor Felix Tietche was born in Bakassa, in the sub-district of Bana, Upper Nkam division in the West Region of Cameroon on 21 November 1952 to the late Kamaha Prosper and Mrs Kuissu Marthe and died on 16 May 2009 in Yaounde. His early life was characterized by discipline but also a great deal of warmth, family love, affection and care. Felix attended the Government primary school of New-Bell in Douala (Cameroon) where he obtained his first school leaving certificate in 1964. He was then admitted to Montaigne secondary school, then to Liberman high school- a school being built on a foundation of both high academic standards and discipline.

Felix Tietche enjoyed the intellectual stimulation of this environment and immersed fully himself in the social milieu, playing keyroles in many students clubs. Felix was academically outstanding and obtained one of the best results in his O-levels and later in his A levels.

After his A levels, he was admitted in the very selective Cameroon Medical School-called CUSS at the time-where he obtained his Doctorate of Medicine in 1977 with distinction. As a member of the first batch of the Cameroon School of Medicine, he set standards which successive batches would strive to emulate. From 1978 to 1984, he served as a medical doctor in several district hospitals in Cameroon. He later won a scholarship that enabled him to attend the University of Tours Medical School in France, where he obtained all his certificates in Pediatrics with a special interest in the newborn. His last research work was focused on the umbilical cord infections as a main cause of neonatal morbidity and mortality in which he tried to demonstrate that the quality of dressing of the umbilical cord at the Chantal BIYA Mother and Child Center in Cameroon could reduce infections and improve survival of neonates.

Professor Felix Tietche was a fine scientist of exceptional rigor and self-discipline, virtues which propelled him to the pinnacle of scientific distinction as a professor of Pediatrics, and a renowned specialist of neonates in the developing world. He was a member of several national and international professional and academic societies. As Director of the Chantal BIYA Mother and Child Center in Cameroon, Professor Felix Tietche commanded respect and has left an enduring legacy of landmark contributions in the management and infection control in neonatal services.

I first met Felix in the 1990's in my capacity of principal investigator of the first ever multicenter study in Cameroon on mother-to-child transmission of HIV. This study demonstrated an increased prevalence of HIV and other sexually transmitted diseases, clarified the mechanisms of the passage of the virus through the placental barrier and contributed to the implementation of policies aimed at curbing the spread of AIDS in Africa as a whole. We were later colleagues when I joined the Faculty of Medicine and Biomedical Sciences of the University of Yaounde I in 2004. Needless to say that Felix was such a friend and a very distinguished colleague. He was indeed one of those very few people who care to listen to the illnesses, whispering the tales of many voiceless in Africa. There is no doubt that Africa has lost one of its intellectual giants. Felix is survived by a wife, Francoise, four children namely Brunel, Sonia, Kevin and Lyne, many family members, and hordes of friends in the world of science, medicine, and academia who will mourn him.

